# Validation of a Mobile App-Based Visual Analog Scale for Appetite Measurement in the Real World: A Randomized Digital Clinical Trial

**DOI:** 10.3390/nu15020304

**Published:** 2023-01-07

**Authors:** Yong Zhu, John E. Blundell, Norton M. Holschuh, Ross McLean, Ravi S. Menon

**Affiliations:** 1 Bell Institute of Health and Nutrition, General Mills, Minneapolis, MN 55427, USA; 2 Appetite Control and Energy Balance Research Group, University of Leeds, Leeds LS2 9JT, UK; 3 Statistics and Data Science, General Mills, Minneapolis, MN 55427, USA; 4 Schlesinger Group, Nashville, TN 37203, USA

**Keywords:** appetite, visual analog scales, digital clinical trial, validation

## Abstract

There has been no validated digital tool for measuring appetite with a visual analog scale (VAS) through a mobile app using participants’ smart phones for data collection in virtual settings. To fill the gap, we developed a digital VAS and conducted a digital cross-over clinical trial by comparing appetite responses measured by this digital tool versus paper-based VAS in 102 participants in a free-living environment. Participants consumed either a 230 or 460 kcal breakfast in randomized order in two virtual sessions, and their appetite was measured over the next 4 h using both tools. The results revealed no significant difference in hunger, fullness, satiety, or desire to eat measured by digital and paper VAS. Paper VAS resulted in a higher prospective consumption score than digital VAS; the difference (1.1 out of 100 points) was statistically significant but not practically relevant. Bland and Altman analysis also indicated consistency in the results from the two methods. In conclusion, digital VAS on a smart phone is a validated tool for appetite measurement in the real world; it provides a new way for researchers to leverage participants’ mobile devices for appetite data collection in digital trials.

## 1. Introduction

Designing foods with greater satiating properties is a potential approach to address increased consumer need for weight management. To assess satiating properties of food or to measure human appetite, certain tools have been developed and validated in laboratory settings. Typically, appetite is measured with paper and pen using self-reported questions with responses captured by lines or Likert scales; although unipolar unstructured line (visual analog scale, VAS) is the most widely used method to measure appetite in clinical trials [1]. In this case, surveys with appetite questions, such as hunger, fullness, satiety, desire to eat, and prospective consumption, can be administered using a piece of paper with responses captured using a 100 mm horizontal line with opposite statements anchored on each end (e.g., not at all, extremely). Participants are instructed to draw a vertical marker on the horizontal line, and the distance from the left end to the position marked by the participant is measured by researchers and recorded.

Over the past two decades, various portable devices have been developed and validated for appetite measurement, such as the Newton-based electronic appetite rating system (EARS) [2,3], Palm-based EARS [4], EARS II with iPAQ [5], wristwatch-based EARS [6], Dell Pocket PC EARS [7], and iPad Mini-based EARS [8]. These are specialized devices provided to participants for use in research studies, either in laboratory settings or in free-living conditions. The use of portable devices offers the flexibility of measuring appetite in any place at any time, while providing a way of checking compliance since the entered appetite data are time-stamped. Nonetheless, these portable devices can be cost-prohibitive for use in clinical trials with a large number of participants. Additionally, participants need to return the devices at the end of the study for data download by researchers, which can be a burden for both parties.

Digital clinical trials have gained increased attention during the COVID-19 pandemic as researchers explore methods to virtually conduct clinical trials. This involves digital technology for execution of a clinical trial with participant recruitment, data collection, and analytics [9]. With regards to appetite research, the widespread use of smart phones in recent years now offers the possibility of “Bring Your Own Device” as another way to collect appetite data in the real world, while participants do not have to physically visit a research facility, unlike in a traditional clinical study. This may reduce the cost of research studies by eliminating the use of researcher-supplied devices and related logistics of shipping or returning of the devices. It may also improve the efficacy of data collection and data quality since researchers may monitor data in real time.

To the best of our knowledge, there has been no validated digital tool for measuring appetite using VAS through a mobile app with participants using their own smart phones in remote settings. Nonetheless, development and validation of such tools are of particular importance for future research applications, considering potential benefits such as reduced research cost, reduced burden to participants and researchers, convenience for use in large-scale digital clinical trials or observational studies, and improved participant engagement and data quality.

The objective of our study was to develop and validate a mobile app-based VAS for appetite measurement in digital clinical trials. Validity was assessed by comparing a newly developed digital VAS with the traditional paper and pen-based VAS for appetite measurements.

## 2. Materials and Methods

### 2.1. Study Design

The study was a randomized digital cross-over trial. Participants received test materials by shipment, installed a mobile app for data collection, and virtually completed two test sessions. Test sessions were separated by a 7-day washout period and participants were given the option to complete a session the next day if not available on the original test date. The informed consent form was signed online before participation. The study was registered at ClinicalTrials.gov (NCT05287516) and the study protocol was reviewed and approved by the Solutions Institutional Review Board (protocol #2022/03/11).

### 2.2. Participants

Participants were recruited by an email sent to panelists from a consumer research panel database. The email provided an outline of the study with a link to an online pre-screening survey. Participants who passed the pre-screening survey were directed to review the informed consent form online and were provided with contact information if they had questions about the study before they decided to participate. To conceal the primary objective of the study, participants were informed that the study was being conducted to evaluate their appetite response to breakfast biscuits. Participants who signed the informed consent form online were directed to complete a formal online screening survey to determine their eligibility for the study. The inclusion criteria for the study were: healthy adults aged 18–70 years, with a body mass index between 20.0 and 29.9 kg/m^2^ based on self-reported weight and height, who understood the study procedure and were willing to follow the study instructions. The exclusion criteria included: pregnant or lactating women; those with known food allergies, sensitivity, or intolerance to any food or food ingredients; those participating in another clinical trial or taking medications affecting appetite, metabolism, or blood pressure; the presence of acute diseases or infection; diagnosis of chronic diseases or eating disorder; restrained eaters as assessed by the Dutch Eating Behavior Questionnaire [10]; those who lost or gained 5 pounds or more in the past 3 months, or on a weight loss diet, or undergoing intermittent fasting; those with COVID-19 infection in the past 3 months.

### 2.3. Mobile App for Data Collection

**Figure 1:**
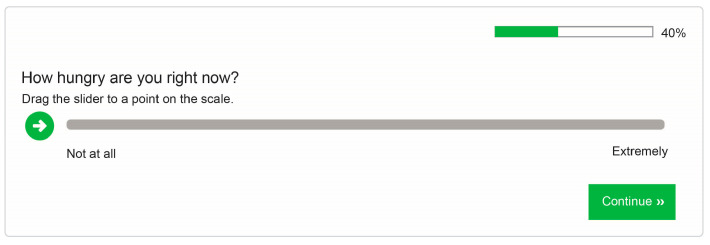
A digital visual analog scale to measure hunger in participants.

The Over the Shoulder^TM^ (OTS) mobile app was used for data collection. Participants were instructed to install the OTS app and complete tutorial assignments to familiarize themselves with various functions of the app prior to the study. The app was programmed to send push notifications to participants each time a survey was needed or for instructions or reminders; data were collected through surveys in the app. For the appetite survey in the app, participants were instructed to set the orientation of their smart phone to landscape mode before the appetite survey questions were displayed. An error message would be displayed if portrait mode was detected, and the appetite survey questions would not be shown until the smart phone was held in landscape mode. The appetite survey included 5 questions related to hunger, fullness, satiety, desire to eat, and prospective consumption [1]. Another question on alertness was also added to distract participants from the appetite questions. The 5 appetite questions are the same questions used in paper-based appetite surveys with VAS. To digitally capture the response, a horizontal line was displayed underneath the question with opposite statements (“not at all” and “extremely”) displayed at each end. Participants were instructed to drag the slider with their finger to a position on the horizontal line. The responses were digitally captured as a numeric value between 0 and 100 depending on the position marked by the participants on the digital scale, with 0 indicating the left end of the scale and 100 indicating the right end of the scale. Figure 1 illustrates the digital VAS for the question on hunger, as an example.

### 2.4. Test Food

BelVita Cinnamon Brown Sugar Breakfast Biscuits (Mondelez International, Chicago, IL, USA) were used in the study. The Nutrition Facts Panel of the product declared that each serving consisted of 4 biscuits with 230 kcal, 36 g of carbohydrates, 8 g of fat, and 4 g of protein. Participants were instructed to consume one serving (230 kcal) of the biscuit in one session, and two servings (460 kcal) of the biscuit in another session, in randomized order. Eight ounces of water were consumed with the biscuits in both sessions.

### 2.5. Research Procedure

Eligible participants were followed up with instructions to install the OTS app and answer questions related to their smart phones, such as manufacturer and model information, and they were asked to provide a shipping address. Study packages were shipped to participants, with test foods and materials including BelVita biscuits, a measuring cup, a ruler, paper appetite surveys, and return envelopes. Upon receiving the package, participants were asked to take a picture of the items received and upload the picture using the OTS app to confirm the items were received without damage. Participants were also asked to complete a paper appetite survey followed by uploading a picture of the completed paper survey, as well as a digital appetite survey, both using the OTS app, as a practice prior to the study. When completing the digital appetite survey, participants were also asked to use a ruler to measure the length of the digital VAS displayed on the screen of their smart phone and report the data to the study team via the OTS app.

On the day prior to the first test session, participants were reminded to avoid heavy physical activity and alcoholic drinks and to maintain their normal diet. Later that day, participants were reminded not to consume any food or beverage, except water, after 10:00 p.m. On the next day, participants were instructed to prepare the test food for breakfast at 7:50 a.m., which included 1 or 2 servings of the biscuits with 8 ounces of water using a measuring cup, take a picture of the breakfast, and upload it using the OTS app. At 8:00 a.m., participants were instructed to complete both a digital and paper appetite survey, in random order, and start eating breakfast. They were asked to finish the entire breakfast in 15 min. Immediately after breakfast, participants were asked to complete another set of digital and paper appetite surveys. Subsequently, participants were asked to not eat or drink anything in the next four hours and avoid moderate or heavy physical activity, while not reading books, watching TV, or browsing the internet for any content related to food. Another set of digital and paper appetite surveys was completed 30, 60, 90, 120, 180, and 240 min after breakfast. Each time, the order of the digital versus paper appetite survey was randomized by asking participants to randomly choose from the two options in the app. After completing a paper survey, participants were asked to upload a picture of the completed survey using the OTS app and then immediately place the completed survey in the designated return envelope.

The procedure for the second test session was similar to that of the first test session, except that a different serving of biscuit was consumed and a final survey about the participants’ experience was administered. The survey collected information on preference for paper versus digital VAS and ease of use for each method using a 5-point scale. After the final survey, participants were debriefed and informed that the primary goal of the study was to compare the digital vs. paper VAS for appetite measurements. Participants were asked to return the completed paper appetite surveys to the study team by mail.

### 2.6. Sample Size

The use of VAS for appetite measurement would require 18 subjects to detect 10% differences in satiety responses using the cross-over design [11]. Power calculation from a previous analysis revealed 20 participants would be sufficient for a validation study [12]. Similarly, a recent review examined 140 appetite studies published in 2016 and reported that sample sizes for cross-over studies varied from 4 to 117, with 20 being the median sample size [13]. The present study recruited 130 participants, considering a potentially higher variability and drop-out rate in the real world, while also testing the feasibility of a relatively large sample size in digital clinical trials.

### 2.7. Statistical Analysis

Data from the paper and digital appetite surveys were analyzed using a mixed model repeated measures ANOVA with baseline values as a covariate for treatment effect (230 kcal vs. 460 kcal), method effect (paper vs. digital), session effect (first vs. second session), time effect (0–240 min), treatment by time interactions, treatment by method interactions, time by method interactions, and treatment by time by method interactions. Agreement between the paper and digital appetite surveys were analyzed using the Bland and Altman method, a diagnostic analysis for comparison of quantitative measurements from two methods [14,15]. A sensitivity analysis on the appetite data was conducted by excluding sessions with appetite survey response submissions that were more than 10 min late, on average, after receiving the notification for appetite survey through the app. Ease of use for both tools was compared by paired t-test. Statistical analysis was performed using SAS 9.4 (SAS Institute, Cary, NC, USA). A value of *p* < 0.05 was considered to be significant.

## 3. Results

### 3.1. CONSORT Flow Diagram and Charecteristics of Participants

**Figure 2:**
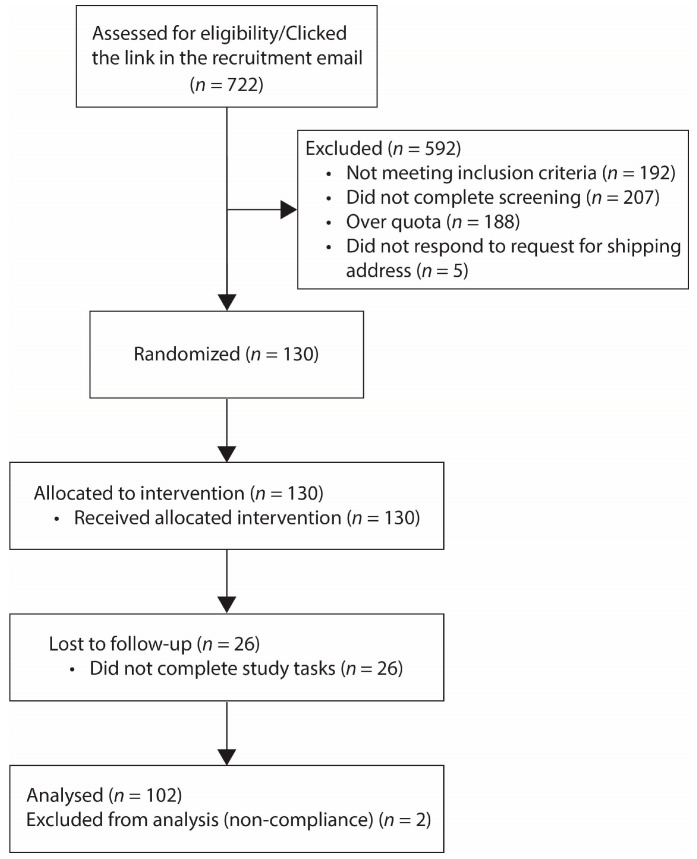
CONSORT flow diagram.

Figure 2 presents the CONSORT flow diagram of the study. There were 722 participants who clicked the link in the recruitment email. 130 eligible participants were enrolled, and they received instructions for installation of the study app by email and the study package by mail. A total of 104 participants completed the study. Review of the time of appetite data submission revealed 2 participants who completed most of their appetite surveys around the same time later in the day, and they were excluded from the analysis. Therefore, our primary analysis included data from 102 participants.

The characteristics of the 102 participants are presented in Table 1. Their age was 40.0 ± 0.9 years, their BMI was 24.5 ± 0.3 kg/m^2^, 78% were female, and 58% were non-Hispanic white participants.

### 3.2. Appetite Data

**Figure 3:**
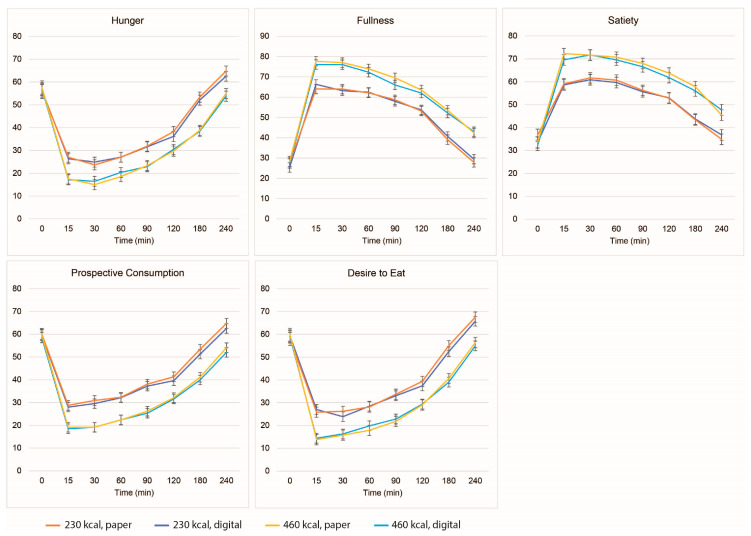
Hunger, fullness, satiety, prospective consumption, and desire to eat (least squares mean ± standard error) following consumption of 230 or 460 kcal breakfast, measured by paper-based or digital visual analog scales.

The appetite responses following consumption of a 230 or 460 kcal breakfast as measured by paper or digital VAS are presented in Figure 3. There was a significant main effect of treatment, as expected. The 460 kcal breakfast resulted in lower hunger, prospective consumption, desire to eat, and higher fullness and satiety than the 230 kcal breakfast (*p* < 0.0001 for all). There was no significant main effect of measurement method on hunger, fullness, satiety, and desire to eat (*p* > 0.05 for all); however, the effect on prospective consumption was significant (*p* = 0.0115), with the paper appetite survey resulting in a higher value than the results from the digital VAS (36.0 ± 1.6 vs. 34.9 ±1.6). As expected, a significant main effect of time was found on each outcome (*p* < 0.0001 for all) since appetite ratings changed over time during the postprandial period. Most of the two-way interactions were non-significant and the three-way interaction was not significant for any appetite outcome in the study. The results from 91 participants in the sensitivity analysis were similar to the results from the primary analysis, except that the main effect of measurement method on prospective consumption was no longer significant (*p* = 0.0941).

### 3.3. Bland and Altman Analysis

**Figure 4:**
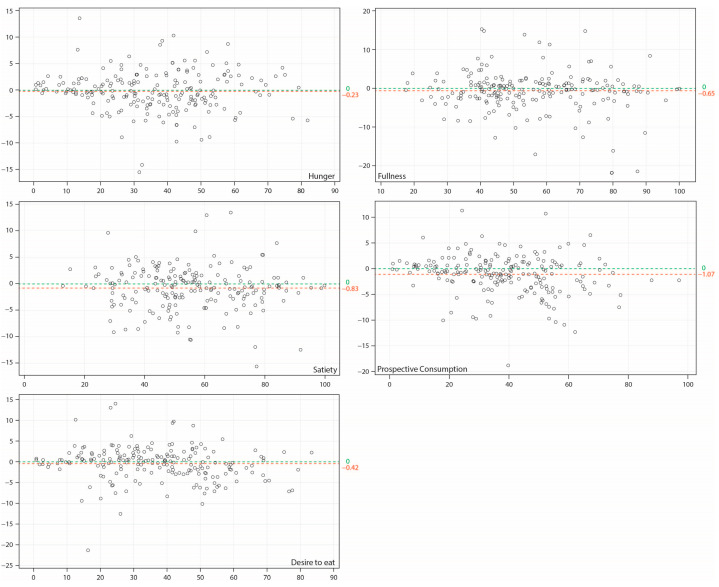
Bland and Altman plots for digital vs. paper visual analog scales for appetite measurement. The vertical axis refers to the mean difference in an appetite rating between digital and paper VAS; the horizontal axis refers to the mean of digital and paper VAS for that appetite rating. The green line indicates an ideal situation where there is zero difference between values measured by digital and paper VAS. The red line indicates the mean difference between values measured by digital and paper VAS.

The results from the Bland and Altman analysis on appetite ratings are presented in Figure 4. These plots represent the mean difference in each appetite rating between digital and paper VAS versus the mean of the appetite rating from digital and paper VAS in each test session for each participant. The mean difference between digital and paper VAS was −0.2 for hunger, −0.7 for fullness, −0.8 for satiety, −1.1 for prospective consumption, and −0.4 for desire to eat, which were negligeable on a 100-point scale. Variability in the difference for each appetite rating was approximately constant across the range, except for very low and very high mean values, which were expected due to a smaller number of participants giving very low or very high scores. There was no discernible pattern in these plots, suggesting that digital VAS provided equivalent measurements to paper VAS.

### 3.4. Participants Experience

Participants rated a similar score on ease of use for both paper and digital VAS (4.6 ± 0.1 for paper VAS vs. 4.7 ± 0.1 for digital VAS, *p* = 0.45). When they were asked their preference, 59% of participants indicated they preferred using digital VAS, whereas 19% indicated they preferred using paper VAS. The remaining 21% of participants had no preference.

## 4. Discussion

The study developed and validated a digital tool for appetite measurement in digital clinical trials while leveraging participants’ own devices. The results indicated that digital VAS can be as sensitive as paper VAS for appetite measurements.

In the primary analysis, it was found that 4 of the 5 appetite ratings did not differ significantly between the two methods assessed; however, prospective consumption was found to be significantly lower when measured by digital VAS than measured by paper VAS. The significant difference may be due to the increased power resulting from the large sample size in the study and the capacity to render very small differences significant. As envisioned, the difference in the least squares means was only 1.1 out of 100 points. A difference at this magnitude is likely to be non-relevant for practical purposes. Moreover, results from the Bland and Altman analysis, as well as results from the sensitivity analysis, further confirmed that both methods provided similar results.

Theoretically, participants may give the same response when they are asked the same question using different tools by recalling their previous response. However, the VAS data was captured on a continuous scale with responses transcribed to a numeric value between 0 and 100 by the researchers, and participants did not directly see the numeric value from the survey they completed. Thus, recalling the exact position they marked on a VAS could be difficult in most cases unless they intentionally measured it. Additionally, to reduce the possibility of participants directly copying the answer from one tool to another tool, the app was programmed such that when the digital VAS was submitted, the data entry could not be retrieved by the participant. Similarly, when the paper VAS was completed, participants were asked to place it into a designated return envelope before completing the digital VAS.

Some recent studies have used mobile phones to collect appetite data using a categorical scale. For example, the PREDICT 1 study used the Zoe study app to collect hunger data using a scale from 0 to 10 in free living conditions [16]. Recently, a digital app called APPetite measuring appetite using a 11-point Likert scale was validated [12]. While both the 11-point Likert scale and VAS have been used to measure appetite in laboratory studies, VAS is more commonly used [1]. Additionally, the use of 100 points in VAS may provide a higher level of sensitivity given that the responses can vary over a broader range than in an 11-point Likert scale, extrapolating conclusions from a study that compared line and categorical scales for measurement of flavor intensity and taste [17]. We could not find any previous study that directly compared VAS with categorical scales for appetite measurement; this could be an interesting question for study in the future. As such, VAS is likely to be more versatile than a Likert scale and certainly more consistent with appetite measurements in research over the last several decades. The new digital tool now offers another and potentially superior platform for appetite measurement in future nutrition studies.

One potential technical challenge in developing a digital VAS with participants’ own devices is that the length of the digital scale displayed on the screen may vary depending on the screen size of a smart phone or the orientation of the device (whether the phone is held in portrait or landscape position). This may not be an issue for responses captured by a 11-point Likert scale since these are essentially multiple choice questions displayed on the screen. To address the orientation issue, the app was programmed in such a way that the appetite survey could only be displayed and completed when the phone was detected to be held in landscape mode. We chose landscape mode rather than portrait mode because the physical length of a digital horizontal line in that situation would be closer to 100 mm, which is the standard length on paper VAS. Data from our study indicated that 74 participants used the iPhone (models varied from iPhone 8 to iPhone 13 Pro Max) and 28 used Android devices (manufacturers included LG, Samsung, Google, and OnePlus). The length of digital VAS displayed on smart phone screens in this study varied between 85 and 128 mm with a median of 105 mm, and 80% of the length data were between 90 and 110 mm. Visual examination of the appetite data did not reveal any specific data pattern related to the physical length of the scale, indicating that length may not be a critical factor. Indeed, Chaput et al. [18] compared 100 and 150 mm lines for paper VAS and concluded the scales were interchangeable for appetite measurement in response to meals in free living conditions based on the results showing no difference between the two tools.

Compared to clinical trials conducted in traditional research facilities, digital studies conducted virtually may have greater external generalizability since participants can be followed up in the real world. However, this brings the potential issue of compliance. As appetite feelings are time dependent, responding to appetite surveys in a timely manner is crucial to ensure the quality of research data. In our primary analysis, we excluded a few subjects who completed multiple appetite surveys around the same time later in the day. We also conducted a sensitivity analysis by excluding subjects who responded to survey tasks at least 10 min late on average. The reason for not excluding these participants in the primary analysis was because missing data can be imputed using the last observation carried forward method in clinical research. When dealing with missing data, appetite data being late by 10 min could be a better option than replacing the missing data with an earlier appetite response, which was usually 30 min earlier in this study. Eleven percent of participants were excluded based on this criterion in the sensitivity analysis. Our compliance was comparable to a previous study reporting that 82% of responses were received within 15 min after a text was sent to participants to report their appetite in a free-living condition [19].

Our study has several clear strengths. It provides a newly validated digital technology that may be used in future studies with the use of mobile devices from participants in a cost and time efficient manner, thus eliminating the requirement of device shipping and return using existing EARS. Our study also included a large sample size with participants recruited remotely in the US, demonstrating that this new tool can be used in large-scale virtual trials without geographic restrictions for participation. Moreover, the digital VAS was validated for use in various smart phone models available on the market today. Nonetheless, our study has certain limitations. First, test-retest reproducibility was not assessed. The reproducibility of paper VAS and EARS have been shown previously [11,20]. In our study, we chose to test the method in two conditions with different caloric preloads; our results revealed similar appetite data from the digital and paper tools in each condition. Second, the study consisted of healthy adult participants with normal or overweight status. Future studies may be needed for assessment of obese subjects, children, or participants with eating disorders. Lastly, the study app was installed on smart phones; it is unknown whether the same conclusion would be reached if the app were used on tablets or computers with much larger screen sizes.

In conclusion, the mobile app-based digital VAS is a validated and preferred tool for measuring appetite in the real world, compared to the traditional paper-based VAS.

## Figures and Tables

**Table 1:** Characteristics of participants.

Characteristics	Results
Age, years, mean ± standard error	40.0 ± 0.9
Body mass index, kg/m^2^, mean ± standard error	24.5 ± 0.3
Gender, *n* (%)	
Male	22 (22%)
Female	80 (78%)
Race/ethnicity, *n* (%)	
Hispanics	12 (12%)
Non-Hispanic White	59 (58%)
Non-Hispanic Black	16 (16%)
Asian or Pacific Islander	9 (9%)
Others, including multiracial	4 (4%)
Prefer not to answer	2 (2%)

## Data Availability

The data presented in this study are available on reasonable request from the corresponding author. The study was registered in www.ClinicalTrials.gov (Identifier NCT05287516).
